# Post-Traumatic Stress in Adolescence: The Mediating Role of Time Perspective Between Trauma Exposure, PTSD Symptoms, and Cannabis Use

**DOI:** 10.3390/ejihpe15090177

**Published:** 2025-09-01

**Authors:** Alexander Pütz, Gerhard Hapfelmeier, Alexandra Martin, Stephan Bender, Marco Walg

**Affiliations:** 1 Clinic for Pediatrics and Adolescent Medicine, Sana-Klinikum Remscheid, 42859 Remscheid, Germany; alexander.puetz@sana.de; 2 Department of Child and Adolescent Psychiatry and Psychotherapy, Sana-Klinikum Remscheid, Burger Str. 211, 42859 Remscheid, Germany; gerhard.hapfelmeier@sana.de; 3 Department of Clinical Psychology and Psychotherapy, University of Wuppertal, 42119 Wuppertal, Germany; martin@uni-wuppertal.de; 4 Department of Child and Adolescent Psychiatry, Psychosomatics, and Psychotherapy, Faculty of Medicine and University Hospital Cologne, University of Cologne, 50937 Cologne, Germany; stephan.bender@uk-koeln.de

**Keywords:** trauma, post-traumatic stress disorder, adolescence, time perspective, cannabis

## Abstract

Post-traumatic stress symptoms (PTSSs) are associated with an imbalanced time perspective (TP) as well as with unhealthy substance use. Although neurodevelopmental processes during adolescence may impact PTSS, most etiological models of post-traumatic stress disorder (PTSD) are derived from studies with adults and neglect possible distinctive characteristics in adolescent PTSD. This study examined possible direct and mediated relations between exposure to potentially traumatic experiences (EPTEs) and PTSS with TP as a potential mediator. In addition, the study investigated the direct and indirect effects of EPTE, TP, and PTSS on cannabis use. The aim of this study was to investigate whether findings from adult studies can be transferred to adolescent PTSD. One hundred and five patients between 14 and 20 years of age were recruited from child and adolescent psychiatric units. They answered questionnaires to assess EPTE, PTSS, TP, and cannabis use. Participants with clinically relevant PTSS showed imbalanced TP with a high orientation to negative past and a low orientation to positive past and future. Higher EPTE was associated with higher level of PTSS, but TP mediated the level of PTSS in this relation. PTSS predicted the frequency of cannabis use. It may therefore be beneficial to consider TP in PTSD therapy with the aim of reducing deviation from balanced TP and PTSS and preventing comorbid substance use.

## 1. Introduction

### 1.1. Trauma and Post-Traumatic Stress in Adolescents

Approximately 60% of adolescents experience at least one potentially traumatic experience before the age of 18 (McLaughlin et al., 2013). Nevertheless, only a minority of exposed adolescents develop psychiatric disorders (Copeland et al., 2007). According to a meta-analysis, the prevalence of post-traumatic stress disorder (PTSD) in potentially traumatic event-exposed adolescents is around 16% (Alisic et al., 2014). Therefore, most children and adolescents experience remission of post-traumatic stress symptoms (PTSSs) and do not meet the full diagnostic criteria for PTSD. A recent study from Germany showed that female adolescents appear to be at higher risk for potentially traumatic experiences and more serious PTSS than male adolescents (Emmerich et al., 2025). A study from Switzerland (Landolt et al., 2013) confirmed that female gender is a risk factor for PTSD, but in contrast showed that female adolescents do not generally experience more potentially traumatic events. The results indicated that male and female adolescents reported different types of events.

Several etiological models of PTSD have been proposed, but the underlying mechanisms that determine whether clinically significant PTSSs develop and persist after exposure to potentially traumatic experiences (EPTEs) are still not fully understood (Lebens & Lauth, 2016). In etiological terms, there is evidence for unique neurodevelopmental processes during adolescence that may impact PTSS (Cisler & Herringa, 2021). Despite the phenotypical similarity, adults and adolescents with PTSD show differences in clinical manifestation: impulsivity, risk taking, and (auto-) aggressive behaviors are more typical in adolescents (Hughesdon et al., 2021; Layne et al., 2014). Nevertheless, there seems to be an overreliance on models derived from adult PTSD studies and a lack of research that specifically investigates PTSD in adolescence.

### 1.2. Time Perspective Theory

The time perspective theory is a more recent and innovative theoretical framework to partially explain the pathogenesis and maintenance of PTSS (Sword et al., 2015). Zimbardo and Boyd defined time perspective (TP) as an unconscious cognitive process that assigns all social and non-social experiences to distinct temporal categories called time dimensions (Zimbardo & Boyd, 1999). TP refers to the personal style individuals mentally partition their experiences into the temporal categories of past, present, and future. These personal orientations influence individuals’ thoughts, emotions, and behaviors. Zimbardo and Boyd (1999) distinguish at least five time dimensions: *Past Negative* (a pessimistic, regretful view of the past), *Past Positive* (a nostalgic, warm view of the past, often linked to tradition and fond memories), *Present Fatalistic* (a current belief that life is controlled by fate, associated with feelings of helplessness and resignation), *Present Hedonistic* (a pleasure-seeking, impulsive focus on the moment), and *Future* (a goal-driven mindset that values planning, typically linked to delayed gratification and long-term rewards). These five dimensions can be assessed using the self-report instrument Zimbardo Time Perspective Inventory (ZPTI; Zimbardo & Boyd, 1999).

Individuals regard each of these time dimensions to varying degrees, which means every person has an individual TP. A balanced TP corresponds to optimal values in each dimension (Zimbardo & Boyd, 2008) and is characterized by high orientations toward Past Positive and Future, moderate Present Hedonistic, and low orientation towards Past Negative and Present Fatalistic. Deviations from optimal values can be quantified using the indicator Deviation from a Balanced Time Perspective (DBTP) (Stolarski et al., 2011) and its revised version DBTP-r (Jankowski et al., 2020). People with imbalanced TP are more prone to maladaptive outcomes, including poor physical and mental health (Stolarski et al., 2020), while a balanced TP seems to have a protective impact (Matthews & Stolarski, 2015).

### 1.3. Time Perspective Theory Applied to Post-Traumatic Stress

People who suffer from PTSS typically show an imbalanced TP characterized by high Past Negative and Present Fatalistic and low Past Positive and Future (Zimbardo et al., 2012). In this vein, prior studies have found positive correlations between DBTP and PTSS (Zhang & Ye, 2022; Stolarski & Cyniak-Cieciura, 2016) and between DBTP and EPTE in adult samples (Tomich et al., 2022; Tomich & Tolich, 2021). The TP theory assumes that EPTE and the negative interpretation of past experiences lead to a high orientation toward Past Negative (Zimbardo et al., 2012). Consequently, people with PTSS tend to interpret past events as negative or focus on adverse experiences rather than positive ones. This concept is compatible with established models of PTSD, which suggest that affected individuals are locked in the past (Ehlers & Clark, 2000). In addition, those affected often experience a sense of current threat (Meiser-Stedman, 2002), helplessness (Panagioti et al., 2015), and low locus of control (Atilola et al., 2021), which is reflected in high orientation toward Present Fatalistic (Zimbardo & Boyd, 1999). Another common symptom of PTSD is a sense of a foreshortened future (Kleim et al., 2013). As simulations of future events are primarily based on past experiences (Schacter et al., 2007), EPTE may result in a negatively biased vision of the future and low orientation toward Future in people with PTSS (Zimbardo et al., 2012). To our knowledge, there is currently no data on the relationship between PTSS and DBTP in adolescents. One study examined TP in adolescent refugees with heightened PTSS and found that they showed imbalanced TP profiles in accordance with the TP theory (Walg et al., 2020b).

Several potential mediators between EPTE and PTSS have been examined to better understand the link between them (Hosseini Ramaghani et al., 2019), with TP being one possible mediator. In a recent study, Tomich et al. demonstrated that the association between cumulative EPTE and PTSS was partially mediated by DBTP in a sample of adults (Tomich et al., 2022). Higher EPTE led to greater DBTP, which resulted in higher levels of PTSS.

### 1.4. Associations Between Post-Traumatic Stress, Time Perspective and Cannabis Use

Adolescents suffering from PTSS are at high-risk for unhealthy drug use (Heradstveit et al., 2019). In clinical studies, the number of potentially traumatic experiences and PTSS severity was positively associated with both the number of psychoactive substances used and with substance use severity (Basedow et al., 2020; Donbaek et al., 2014; Patel et al., 2024). A major hypothesis is that individuals with PTSS use substances to self-medicate and attenuate symptoms (Khantzian et al., 1999). In line with this, a longitudinal study from 2024 showed that PTSS predicted the number of substances used in adolescence (Patel et al., 2024).

Recently, cannabis use has been legalized for the adult population in Germany (Deutscher Bundestag, 2024). Until then, cannabis was the most popular illicit drug (Manthey et al., 2024; Orth & Merkel, 2022) and remains a widely used drug among adolescents across Europe (European Monitoring Centre for Drugs and Addiction, 2024). A few studies have examined the co-occurrence of PTSS and cannabis use in adolescents and found that PTSS and cannabis use were significantly correlated (Hicks et al., 2022). Substance use and TP also seem to be associated. As suggested by Zimbardo and Boyd (1999), some studies have found that present and future TP are related to cannabis use (Apostolidis et al., 2006; Barnett et al., 2013), whereas a balanced TP was associated with lower overall substance use in adult samples (Finan et al., 2021).

### 1.5. Aim of This Study and Hypotheses

This study examined the TP profile of adolescents with clinically relevant PTSS. This study is the first to investigate the possible associations between PTSS, DBTP, and cannabis use in combination with a clinical sample of adolescents. To address the lack of research, this study also examined whether previous findings from adult samples could be transferred to the adolescent developmental stage.

**Figure 1:**
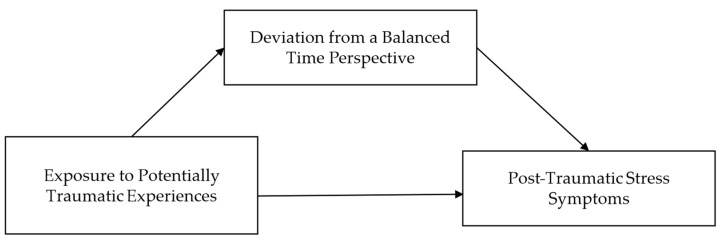
Proposed mediation model linking Exposure to Potentially Traumatic Experiences and Post-Traumatic Stress Symptoms via Deviation from a Balanced Time Perspective.

**Figure 2:**
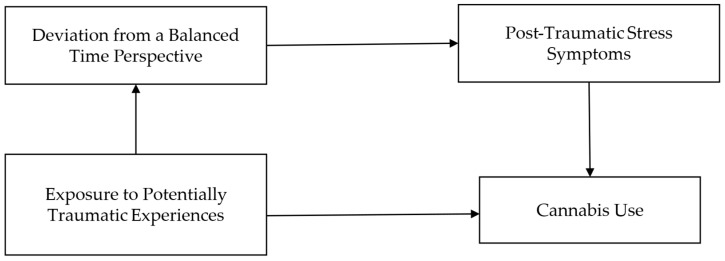
Proposed mediation model linking Exposure to Potentially Traumatic Experiences and Cannabis Use via Deviation from a Balanced Time Perspective and Post-Traumatic Stress Symptoms.

Based on the current state of theory and research we hypothesized the following:

- Adolescents with PTSS show an imbalanced TP characterized by high orientations towards Past Negative and Present Fatalistic and low orientation towards Past Positive and Future.
- The relationship between EPTE and PTSS is mediated by DBTP analogous to the model proposed by Tomich et al. (2022) (see Figure 1).

- The relationship between EPTE and cannabis use is mediated by PTSS and DBTP serially (see Figure 2).

## 2. Materials and Methods

### 2.1. Participants and Procedure

Participants were recruited from child and adolescent psychiatric outpatient and day clinics in Germany. Recruitment took place at the beginning of the patients’ respective treatments at first contact. The initial consultation in clinics lasted at least one hour and included not only the assessment of symptoms by a clinical interview, but also the assessment of psychopathological findings according to the manual for assessment and documentation of psychopathology in psychiatry (Broome et al., 2018). As the assessment for this study was conducted immediately after the initial consultation in clinics, no confirmed diagnoses were available at the time. The patients either had suspected diagnoses from a medical respective psychotherapeutic practice or received suspected diagnoses after the initial consultation in the outpatient clinic or day clinic.

All patients between 14 and 20 years of age were invited to participate in the study. Exclusion criteria included psychotic symptoms, dyslexia, or below-average cognitive abilities based on clinical impression during an initial consultation in clinics and upon review of school report cards. The exclusion criteria were chosen with the intention of ensuring that all participants could complete the study survey independently and adequately to ensure the highest possible data quality. Questions about EPTE, such as sexual violence, can trigger intense feelings of shame. It was likely that participants would avoid asking an unknown clinic employee for explanations if they had difficulties understanding such shame-triggering topics. In order to avoid biases of this kind as far as possible, subjects with characteristics that could potentially impair text comprehension were not included in the study.

The study was carried out cross-sectionally. Consent to participate in the study was obtained from all participating adolescents and their legal guardians. The patients completed a one-time online survey using the software *LamaPoll* 2025 under the supervision of a clinic employee. Data were collected from November 2023 to August 2024. Participants were recruited after the study received a positive vote from the Research Ethics Committee of the North Rhine State Chamber of Physicians (ÄkNo), Germany in April 2023 (code: 2023048).

### 2.2. Measures

#### 2.2.1. Child and Adolescent Trauma Screen–2

The German version of the *Child and Adolescent Trauma Screen–2* (CATS-2; Sachser et al., 2023) is a self-report questionnaire that assesses cumulative EPTE and PTSS in children and adolescents according to the ICD-10 (World Health Organization, 2019) and DSM-5 (American Psychiatric Association, 2013). Cumulative EPTE assessments rely on a checklist of 15 different potentially traumatic events in which respondents indicate whether they have experienced each event in the past or not on a dichotomous scale. The resulting sum score of 0–15 serves as an indicator of cumulative EPTE. PTSS levels are assessed by 25 items that measure the frequency of PTSS on a 4-point Likert scale (0 = never; 3 = almost always). These items represent the four main symptom clusters of PTSD according to the DSM-5: intrusion, avoidance, negative changes in mood, and cognition and hyperarousal. Sum scores of 25 points or higher indicate elevated PTSS and suspected PTSD (Sachser et al., 2023). The internal consistency of the CATS-2 in this sample was high (α = 0.94).

#### 2.2.2. Zimbardo Time Perspective Inventory and Deviation from a Balanced Time Perspective

A German short version of the Zimbardo Time Perspective Inventory (ZTPI) was used to assess TP (Danner et al., 2019). Each of its five time dimensions is measured by two items on a 5-point Likert scale (1 = very untrue; 5 = very true). Means were computed for every subscale (PP = Past Negative, PN = Past Positive, PF = Present Fatalistic, PH = Present Hedonistic, F = Future). In this sample, Spearman–Brown internal consistencies were r_SB_ = 0.67 for Past Negative; r_SB_ = 0.56 for Past Positive; r_SB_ = 0.45 for Present Fatalistic; r_SB_ = 0.57 for Present Hedonistic; and r_SB_ = 0.62 for F. These values are similar to the calculated reliabilities in the validation study (Danner et al., 2019).

The indicator DBTP-r was calculated using the formula proposed by Jankowski et al. (Jankowski et al., 2020) to quantify TB balance:

$$DBTP-r=\sqrt{{{\left(oPN-ePN\right)}^{2}+(oPP-ePP)}^{2}+{\left(oPF-ePF\right)}^{2}+{\left(oPH-ePH\right)}^{2}+{\left(oF-eF\right)}^{2}}$$

Optimal values are as follows: oPN = 1.00; oPP = 5.00; oPF = 1.00; oPH = 3.40; oF = 5.00 (Jankowski et al., 2020). The empirical scores (e.g., ePN) correspond to the mean values of the five scales. If DBTP-r is equal to 0, TP is fully balanced. The higher the DBTP-r score, the greater the deviation from the optimal balanced time perspective.

#### 2.2.3. Cannabis Use

The frequency of cannabis use was assessed using one item from the *Heidelberger Drug Scale* (Aguilar-Raab et al., 2015): “How often have you consumed cannabis in the last 12 months?”. Responses were scored on a 7-point Likert scale ranging from (0) “never” to (7) “multiple times a day”.

### 2.3. Statistical Analyses

Descriptive data, median comparisons, and correlational analyses were computed with the Package for the Social Sciences (IBM SPSS, version 29.0.2.0) for windows. The macro PROCESS (version 4.2) by Hayes (Hayes, 2022) was used to perform mediation analyses. According to Hayes, the violation of single assumptions of the mediation analysis can be tolerated (Hayes, 2022). Shapiro–Wilk tests indicated that Cannabis use and EPTE were not normally distributed (*p* ≤ 0.001). The time dimensions Past Negative, Past Positive, Present Fatalistic, and Future also exhibited a non-normal data distribution in participants with clinically relevant PTSS (*p* ≤ 0.001). This was supported by the visual examination of the data distribution (histograms and QQ diagrams). Consequently, correlational analyses were carried out non-parametrically using Spearman rank correlations. To compare the balanced TP profile with the TP profile of patients with clinically relevant PTSS, we conducted non-parametrical one-sample Wilcoxon tests. For this purpose, a subsample was drawn that included participants who had a CATS-2 score ≥ 25. Bootstrapping was employed for the mediation analyses to estimate unstandardized regression coefficients, confidence intervals for these coefficients, and additional standardized coefficients as a robust method to cope with the non-normally distributed data (Hayes, 2009). Ten thousand bootstrapping samples were computed for each model parameter. The statistical significance of the model paths was evaluated based on the bootstrapped confidence intervals. If a confidence interval did not contain a 0, the associated effect was considered statistically significant. Effect sizes were assessed according to Ferguson’s conventions (Ferguson, 2016). Given the large difference in subgroup sample sizes, Mann–Whitney U Tests were calculated to assess possible differences between male and female participants. Chi-squared tests were calculated to examine possible associations between gender and cannabis use. Two participants who identified as neither male nor female were excluded due to the small subsample size.

## 3. Results

### 3.1. Sample Characteristics

One hundred and five adolescents between 14 and 19 years of age (*M* = 15.6; *SD* = 1.3) participated in this study. Most of the participants were biologically female (73%). The remaining patients were male (25%) or did not identify as male or female (2%). There was no significant age difference between female (*M* = 15.6; *SD* = 1.3) and male participants (*M* = 15.4; *SD* = 1.4; *U* = 912.5; *p* = 0.489). About 59% of patients were recruited from a day clinic setting and the remaining 41% came from outpatient facilities. Sociodemographic and clinical charactersistics including ICD-10 diagnoses are shown in Table 1. The most common suspected diagnoses were depressive episode (41%), emotional disorder (32%), and attention-deficit/hyperactivity disorder (30%). Approximately 65% of the patients had more than one clinical diagnosis.

### 3.2. Post-Traumatic Stress Symptoms, Deviation from Balanced Time Perspective and Cannabis Use

The mean CATS-2 sum score was 28 (*SD* = 12), which indicated elevated PTSS. Approximately 65% (*n* = 66) exhibited clinically relevant PTSS (CATS-2 score ≥ 25). On average, four potentially traumatic events were reported per patient (*SD* = 2.9). Descriptive data is displayed in Table 2. At least one event was reported by 94% of participants. The female patients in our sample reported higher EPTE (*M* = 4.7; *SD* = 3.1) than male patients (*M* = 3.2; *SD* = 2.2; *U* = 728.0; *p* = 0.037) and female adolescents exhibited higher PTSS (*M* = 31.4; *SD* = 11.1) compared to males (*M* = 18.7; *SD* = 10.1; *U* = 403.0; *p* < 0.001). Approximately 40% of patients (*n* = 42) reported having used cannabis at least once within the last 12 months. Taking the unequal gender ratio in the sample into account, among the adolescents that reported cannabis use, significantly more females (*n* = 36; 85.7%) than expected and respectively fewer males (*n* = 6; 14.3%) than expected were reported by chance (χ^2^ = 4.5, *p* < 0.034, Cramer V = 0.21).

As shown in Table 2, all variables were significantly positively intercorrelated. PTSS and DBTP-r correlated moderately (*r* = 0.60; *p* ≤ 0.001). For cannabis use, significant correlations were found with PTSS (*r* = 0.32; *p* ≤ 0.001), EPTE (*r* = 0.27; *p* = 0.002), and DBTP-r (*r* = 0.23; *p* = 0.006).

### 3.3. Time Perspective Profile

**Figure 3:**
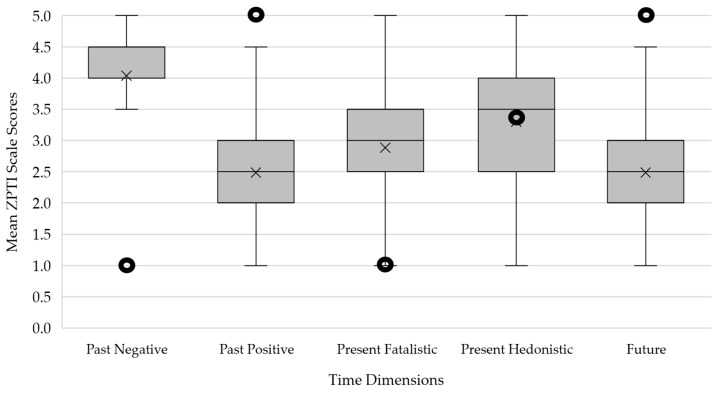
Time Perspective Profile of participants with clinically relevant elevated PTSS (*n* = 66) displayed as boxplot diagrams. Crosses indicate the sample’s mean score, and horizontal lines indicate the median for each time dimension. Punched dots represent the optimal values of the balanced time perspective profile according to Jankowski et al. (2020). ZTPI = Zimbardo Time Perspective Inventory.

As Figure 3 illustrates, participants with clinically relevant PTSS according to CATS-2 showed typical deviations from optimal values in most time dimensions, namely, significantly higher Past Negative (*Z* = 7.14; *p* ≤ 0.001; *d* = 1.91) and Present Fatalistic (*Z* = 7.01; *p* ≤ 0.001; *d* = 1.84), and significantly lower Past Positive (*Z* = −7.10; *p* ≤ 0.001; *d* = −1.91) and Future (*Z* = −7.10; *p* ≤ 0.001; *d* = −1.91). The orientation toward Present Hedonistic did not differ significantly from the optimal value (*Z* = 0.27; *p* = 0.79; *d* = 0.04). Female patients showed higher DBTP-r (*M* = 5.4; *SD* = 0.9) than male patients (*M* = 4.9; *SD* = 1.1; *U* = 711.0; *p* = 0.028).

### 3.4. Mediation Analysis 1: Exposure to Potentially Traumatic Experiences, Deviation from a Balanced Time Perspective and Post-Traumatic Stress Symptoms

**Figure 4:**
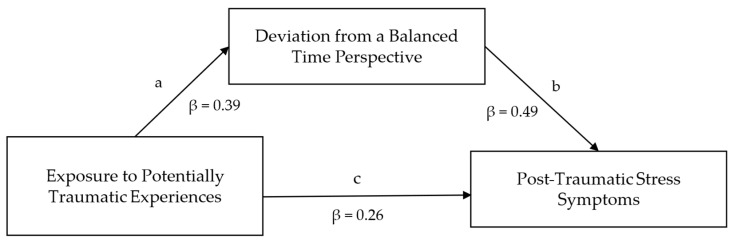
Mediation model linking Exposure to Potentially Traumatic Experiences and Post-Traumatic Stress Symptoms via Deviation from a Balanced Time Perspective. Letters represent the names of the paths. Path coefficients indicate direct effects and are standardized. Dashed lines indicate non-significant direct effects; solid lines indicate significant direct effects.

The mediation model is displayed in Figure 4.

The direct path c from EPTE to PTSS was significant; therefore, EPTE significantly predicted PTSS (B = 1.06; 95% CI [0.42, 1.64]; β = 0.26). EPTE also significantly predicted DBTP (path a; B = 0.12; 95% CI [0.06, 0.18]; β = 0.39). DBTP had a significant direct effect on PTSS (path b; B = 6.48; 95% CI [4.42, 8.61]; β = 0.49). The indirect mediated effect of EPTE on PTSS with DBTP-r as a mediator was also significant (B = 0.81; 95% CI [0.38, 1.34]; β = 0.20), indicating a mediation of DBTP between EPTE and PTSS. In total, the model explained 20.3% of the variance in PTSS.

### 3.5. Mediation Analysis 2: Exposure to Potentially Traumatic Experiences, Deviation from a Balanced Time Perspective, Post-Traumatic Stress Symptoms and Cannabis Use

**Figure 5:**
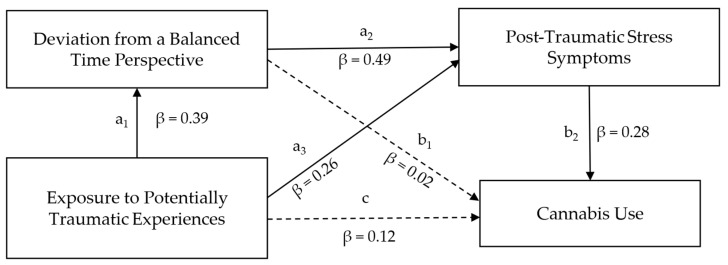
Serial mediation model linking Exposure to Potentially Traumatic Experiences and Cannabis Use via Deviation from a Balanced Time Perspective and Post-Traumatic Stress Symptoms. Letters represent the names of the paths. Path coefficients indicate direct effects and are standardized. Dashed lines indicated non-significant direct effects; solid lines indicate significant direct effects.

The extended serial mediation model is displayed in Figure 5. EPTE did not significantly predict cannabis use directly (path c; B = 0.06; 95% CI [−0.06; 0.20]; β = 0.12), but cannabis use was significantly predicted by PTSS (path b_2_; B = 0.03; 95% CI [0.01; 0.07]; β = 28). There was an indirect effect of EPTE on cannabis use via PTSS (B = 0.05; 95% CI [0.01; 0.10]; β = 0.08). Another indirect effect of EPTE on cannabis use via DBTP-r and PTSS in succession was found (B = 0.06; 95% CI [0.01; 0.13]; β = 0.06). There was no significant indirect effect of EPTE on cannabis use via DBTP-r (B = 0.003; 95% CI [−0.04, 0.06]; β = 0.01). The extended model explained 14.0% of the variance in cannabis use.

## 4. Discussion

This study examined TP as a potential mediator in the link between cumulative EPTE and PTSS in adolescents. Additionally, potential associations with cannabis use were examined.

The results from Tomich et al. (2022) were successfully replicated in this sample of adolescents. This study therefore showed that the proposed model is also applicable to adolescent PTSS. Findings indicate that cumulative EPTE led, as postulated, to a stronger DBTP, which resulted in more severe PTSS. Adolescents with clinically relevant PTSS showed a distorted TP, which is common for individuals with PTSD, particularly high orientation toward Past Negative as well as low orientation toward Past Positive and Future (Zimbardo et al., 2012). People who suffer from PTSS focus on the traumatic experiences in their past. Hence, high scores on Past Negative may reflect the re-experiencing symptoms in PTSD (Ehlers et al., 2004). At the same time, they seem to neglect or deny positive aspects of their past, which manifests as low levels of Past Positive. Low orientation toward Future is also common for individuals with PTSD as they are locked in the negative past (Holman & Silver, 1998). In line with this, a recent study from 2024 pointed out that higher orientation toward Future is associated with post-traumatic growth (Saltzman & Terzis, 2024).

The results have implications for treating adolescent PTSS. Considering TP in diagnostics and therapy may be beneficial. Assessing and balancing TP could augment well-established intervention programs for adolescents with PTSS. Decreasing the orientation toward Past Negative and building a higher orientation toward Past Positive might expedite the reduction in PTSS. Fostering higher orientation toward Future may promote post-traumatic growth. The TP theory has already been applied to clinical interventions for PTSD. Zimbardo, Sword and Sword have developed a TP therapy for PTSD that aims to balance TP, thereby achieving a reduction in PTSS (Zimbardo et al., 2012). Primary studies indicate the effectiveness of this new intervention in adults suffering from PTSS (Sword et al., 2015). Some of these considerations have already been transferred to clinical psychological interventions targeting adolescents. For instance, Walg (2017) developed a short intervention which in part aims to balance the TP of adolescent refugees to improve their trauma-related symptomology. This program succeeded in improving global distress and anxiety symptoms in adolescents (Walg et al., 2020a). The results also highlight the potential significance of balanced TP as a protective factor. Previous studies of adult populations have suggested that a balanced TP is associated with a variety of positive health outcomes (Stolarski et al., 2020). Consequently, prevention strategies aimed at adolescents exposed to potentially traumatic events could also benefit from strategies that balance TP, thereby reducing the risk of PTSD and other trauma-related complications.

In an expansion of the mediation model by Tomich et al. (2022), we showed that the cumulative EPTE had a significant indirect effect on cannabis use, which was mediated by DBTP and PTSS. As postulated, a higher cumulative EPTE presumably led to a less balanced TP, which promoted more severe PTSS, which in turn resulted in more frequent cannabis use among the affected adolescents. There was also a mediated path linking EPTE and cannabis use via PTSS, indicating that higher EPTE led to a more severe PTSS, which resulted in more frequent cannabis use. Apart from these results, we could not confirm a direct effect of EPTE on cannabis use. This finding is contrary to prior studies (Basedow et al., 2020; Donbaek et al., 2014). According to our findings, DBTP does not seem to be a direct predictor of cannabis use in adolescents. This corresponds to prior evidence in adults, which suggested that DBTP and substance use in general are unrelated (Stolarski et al., 2020).

The results are in line with the self-medication hypothesis (Khantzian et al., 1999) and support earlier findings that indicate adolescents with PTSS are at high-risk for unhealthy drug use (Heradstveit et al., 2019) and, cannabis use disorders in particular (Cornelius et al., 2010). Frequent and heavy cannabis use during adolescence is associated with negative impacts on brain health as well as subsequent cognitive impairment and psychiatric complications in adulthood (Levine et al., 2017). Adolescents with childhood trauma and early cannabis use are at a particularly high risk of developing psychotic symptoms (Harley et al., 2010). A review from 2024 pointed out that cannabis use does not improve overall PTSS, but on the contrary increases the risks for worsening PTSS, suicidal ideation, and violent behavior (Rodas et al., 2024). To prevent comorbid cannabis use disorder, giving young people with PTSS access to psychotherapy as quickly as possible is essential. In an era in which the legalization of cannabis is increasingly common, further efforts should be made to make parents, teachers, and social workers aware of the risks of cannabis use in sensitive developmental periods as well as to establish programs to prevent unhealthy drug use.

The relations found between EPTE, PTSS and cannabis use should also be considered in drug counseling and the treatment of substance use disorders. The findings underline the importance of accurately assessing for PTSS among individuals with unhealthy substance use. There is evidence that PTSD is often overlooked in clinical evaluations of adolescents with substance use disorders (Cornelius et al., 2010). If the underlying PTSD goes unrecognized, patients with substance use disorders may not receive adequate treatment.

### Strengths and Limitations

Similar to the results from Tomich et al. (2022) regarding adult PTSS, conclusions about causality are limited due to the cross-sectional nature of the study. We can only presume the underlying mechanism. Future research should investigate the impact of potentially traumatic experiences on TP and its influence on PTSS development longitudinally and prospectively to allow for more valid conclusions.

The sample size is rather small and unbalanced in terms of gender distribution with predominantly female participants, which limits the generalizability of the findings. Therefore, possible gender differences in all relevant variables were examined. Female patients had a significantly less balanced time perspective than male patients. This finding contrasts with previous studies of mostly healthy adult samples, which found no gender-related differences in DBTP (Fuentes et al., 2022; Macałka et al., 2022). Future large-scale studies should therefore investigate gender differences in TP in adolescent clinical samples, particularly in those suffering from PTSD. Female patients also reported more potentially traumatic experiences and higher PTSS than male patients. The higher cumulative EPTE in female adolescents in this sample is contrary to prior evidence (McLaughlin et al., 2013) but may explain the less balanced TP (higher DBTP) and consequently higher PTSS in female adolescents compared to males. Although approximately 1.75 times more young men than young women consume cannabis in Germany (Job & Brieske, 2025), there were significantly more female participants who reported cannabis use than males in the current study. As the expanded mediation model of this study suggests, the higher EPTE in female participants may have led to a less balanced TP, which may have promoted higher PTSS and resulted in a higher incidence of cannabis use.

Participants were of average intelligence according to the clinical impressions of child and adolescent psychiatrists and psychotherapists with several years of professional experience and according to the school report cards reviewed. No standardized intelligence test was conducted. However, studies indicate that mental disorders, especially PTSD, significantly impair the performance of children and adolescents in intelligence testing (Barrera-Valencia et al., 2017). Therefore, it is questionable whether a measured IQ score would actually be more valid than the clinical impressions and school report cards used for this clinical sample.

The relevant variables of this study are exclusively based on self-report data, which may have promoted biases commonly found in self-report clinical data in adolescent populations (Hughes & Gullone, 2010) and may only partly reflect the patient’s symptomatology (Smith et al., 2019). However, a multi-informant approach does not necessarily lead to more reliable results, especially in the case of internalizing symptoms in clinical samples (De Los Reyes et al., 2015). In particular, the CATS-2 is a highly sensitive, specific and reliable self-report instrument (Sachser et al., 2022).

A major strength of this study is the transfer of TP research in a clinical context to the adolescent phase of development. Although this study replicated results from prior studies conducted on adults, it falls short of implementing a developmental framework. TP has been conceptualized as a developmental and modifiable mechanism that can differentiate between characteristics in different age groups (Mello, 2019). Adolescence is a period of significant brain development and is associated with changes in cognitive and socioemotional functioning (Fombouchet et al., 2023; Yurgelun-Todd, 2007), which may also be reflected in age specific changes in TP (Mello & Worrell, 2006). As part of this ongoing cerebral development, adolescence is characterized by increased impulsivity, reward- and risk-seeking behavior, and deficits in the ability to delay gratification (Collado et al., 2014; Harden & Tucker-Drob, 2011). This, in turn, is reflected in a high Present Hedonistic TP in adolescents as compared to other age groups (Klicperová-Baker et al., 2020; Laureiro-Martinez et al., 2017). In contradiction to this, we did not find elevated levels of Present Hedonism compared to the optimal values of the balanced TP profile. Future studies should place greater emphasis on these developmental aspects to gain a better understanding of the peculiarities of adolescent TP and their possible significance in connection with PTSD and cannabis use.

In the context of the ongoing debate concerning the TP theory and balanced TP, the ZTPI has been criticized in its use among adolescent populations due to the insufficient factorial validity in this age group (Mello & Worrell, 2006; Worrell et al., 2016). Newer revisions of the TP theory seem to provide a promising alternative for TP research in adolescents (Mello & Worrell, 2015). A balanced TP is an intuitive concept that emerged as correlate of various aspects of health, but has been criticized as a simplification in its current form (McKay et al., 2022). Moreover, because it is questionable whether a universal optimal TP profile for all age groups can be found, further research efforts should instead consider using other operationalizations, such as the Adolescent Time Inventory (Mello & Worrel, 2007), and employ different approaches, such as cluster analyses (McKay et al., 2014). This is especially relevant since the psychometric properties of the short form of the ZTPI were moderate to poor in this study, which limited the reliability of the TP assessment.

## 5. Conclusions

The findings show that DBTP mediates the link between EPTE and PTSS in adolescents. Therefore, balancing the TP should be considered in PTSD therapy to reduce DBTP and PTSS. Furthermore, a balanced TP may be an important protective factor during adolescence, increasing resilience in vulnerable groups exposed to potentially traumatic events, as well as the general population. In line with the self-medication hypothesis, adolescents suffering from PTSS are at high risk for unhealthy cannabis use. To prevent comorbid substance use disorder, adolescents with PTSS should be given access to trauma-focused psychotherapy or other types of psychological support as quickly as possible.

## Figures and Tables

**Table 1:** Sociodemographic and clinical characteristics of adolescent patients (*N* = 105).

Age in years *M* ± *SD*	15.6 ± 1.3
Sex *n* (%)	
male	26 (25%)
female	77 (73%)
gender diverse	2 (2%)
Clinical setting *n* (%)	
Day clinic	62 (59%)
Outpatient clinic	43 (41%)
Clinical ICD-10 diagnoses *n* (%)	
Unhealthy use of alcohol (F10.1)	2 (2%)
Unhealthy use of cannabis (F12.1/F12.2)	5 (5%)
Unhealthy use of tobacco (F17.2)	7 (7%)
Unhealthy multiple drug use (F19.1)	1 (1%)
Bipolar affective disorder (F31.1)	1 (1%)
Depressive episode (F32)	43 (41%)
Social phobia (F40.1)	16 (15%)
Specific phobia (F40.2)	2 (2%)
Obsessive-compulsive disorder (F42)	1 (1%)
PTSD (F43.1)	10 (10%)
Adjustment disorder (F43.2)	8 (8%)
Somatization disorder (F45.0)	1 (1%)
Anorexia nervosa (F50.0/F50.1)	3 (3%)
Borderline Personality Disorder (F60.31)	7 (7%)
Asperger’s syndrome	3 (3%)
Attention-deficit/hyperactivity disorder (F90.0/F90.1)	31 (30%)
Mixed disorders of conduct and emotions (F92)	5 (5%)
Emotional disorders (F93)	34 (32%)
Comorbidity *n* (%)	68 (65%)

*Note.* The percentages of diagnoses do not add up to 100% because the majority of patients had more than one diagnosis. The percentages are rounded.

**Table 2:** Descriptive data and non-parametric correlations of the major variables.

	Descriptive Statistics		Spearman Rank Correlations		
	*M*	*SD*	PTSS	EPTE	DBTP-r
PTSS	28.3	12.0			
EPTE	4.3	2.9	0.40 ***		
DBTP-r	5.3	0.9	0.60 ***	0.36 ***	
Cannabis Use	1.1	1.8	0.32 ***	0.27 **	0.23 **

*N* = 105; PTSSs = Post-traumatic Stress Symptoms, EPTEs = Exposure to Potentially Traumatic Experiences; DBTP-r = Deviation from a Balanced Time Perspective-revisited. ** *p* = 0.01; *** *p* ≤ 0.001.

## Data Availability

The data presented in this study are available on request from the corresponding author. The data are not publicly available due to ethical restrictions and data protection according to German law.

## Abbreviations

The following abbreviations are used in this manuscript:

PTSSs	Post-traumatic stress symptoms
TP	Time perspective
PTSD	Post-traumatic stress disorder
EPTEs	Exposure to potentially traumatic experiences
DBTP	Deviation from a Balanced Time Perspective
DBT-r	Deviation from a Balanced Time Perspective-revised version
CATS-2	Child and Adolescent Trauma Screen-2
ZTPI	Zimbardo Time Perspective Inventory
